# List of Accidents, Admitted into the Pennsylvania Hospital, from May 2d to May 16th, 1838

**Published:** 1838-05-23

**Authors:** 


### CLINICAL REPORTS.

*List of Accidents, admitted into the Pennsylvania Hospital, from May 2d to May 16th,* 1838.

One case of lacerated wound of the thick part of the fore-arm, caused by the bursting of a gun; dressed with a poultice and carved angular splint; matter formed beneath the fascia, which was freely opened; now healing. One case of incised wound of the ankle, caused by a cut with an axe; the integuments cut through to the bone, the joint, however, escaped; the posterior tibial artery and tendo Achillis were both divided; vessels secured; wound poulticed; foot extended, and placed in a carved splint; doing well. One case of incised wound of fore-head, of upper lip, division of lower lip, and of portion of tongue; fore-head and upper lip dressed with adhesive plaster; lower lip with hare-lip needle and suture; tongue hooked out of the mouth and stitched; since dead from mania a potu. One case of simple fracture of both bones of the leg, from a fall, dressed with fracture box. One case of fracture of tibia, close to the ankle, caused by a fall; dressed in same manner. One case of deep incised wound of the abdomen, caused by a stab with a dirk; peritonitis followed, treated accordingly, and doing well. One case of compound comminuted fracture of both bones of the leg, caused by being run over by a heavy waggon; sloughing ensued, and, afterwards, mortification, which extended to the groin; dressed with large fomenting poultices, fracture box, full diet, quinine, porter, &c.; attacked with mania a potu; since dead. One case of simple oblique fracture of the lower end of the radius; dressed with two padded splints. One case of simple fracture of the tibia near the ankle, and of fibula near the knee; dressed with fracture box. One case of dislocation of the upper extremity of the radius, to be reported at length in the next number.

Dr. Thomas Stewardson was elected one of the Physicians to the Pennsylvania Hospital, on Monday, 14th of May.

